# A Multi-Center Comparison of 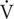
O_2peak_ Trainability Between Interval Training and Moderate Intensity Continuous Training

**DOI:** 10.3389/fphys.2019.00019

**Published:** 2019-02-05

**Authors:** Camilla J. Williams, Brendon J. Gurd, Jacob T. Bonafiglia, Sarah Voisin, Zhixiu Li, Nicholas Harvey, Ilaria Croci, Jenna L. Taylor, Trishan Gajanand, Joyce S. Ramos, Robert G. Fassett, Jonathan P. Little, Monique E. Francois, Christopher M. Hearon, Satyam Sarma, Sylvan L.J.E. Janssen, Emeline M. Van Craenenbroeck, Paul Beckers, Véronique A. Cornelissen, Nele Pattyn, Erin J. Howden, Shelley E. Keating, Anja Bye, Dorthe Stensvold, Ulrik Wisloff, Ioannis Papadimitriou, Xu Yan, David J. Bishop, Nir Eynon, Jeff S. Coombes

**Affiliations:** ^1^School of Human Movement and Nutrition Sciences, The University of Queensland, Brisbane, QLD, Australia; ^2^School of Kinesiology and Health Studies, Queen’s University, Kingston, ON, Canada; ^3^Institute for Health and Sport (iHeS), Victoria University, Melbourne, VIC, Australia; ^4^Translational Genomics Group, Institute of Health and Biomedical Innovation, Queensland University of Technology at Translational Research Institute, Princess Alexandra Hospital, Brisbane, QLD, Australia; ^5^Faculty of Health Sciences and Medicine, Bond University, Robina, QLD, Australia; ^6^K.G. Jebsen Center of Exercise in Medicine, Department of Circulation and Medical Imaging, Faculty of Medicine, Norwegian University of Science and Technology, Trondheim, Norway; ^7^SHAPE Research Centre, Exercise Science and Clinical Exercise Physiology, College of Nursing and Health Sciences, Flinders University, Adelaide, SA, Australia; ^8^School of Health and Exercise Sciences, University of British Columbia, Kelowna, BC, Canada; ^9^Internal Medicine, Institute for Exercise and Environmental Medicine, University of Texas Southwestern Medical Center, Dallas, TX, United States; ^10^Department of Physiology, Radboud University Medical Center, Nijmegen, Netherlands; ^11^Cardiology Department, Antwerp University Hospital, Antwerp, Belgium; ^12^Department of Rehabilitation Sciences – Research Group for Rehabilitation in Internal Disorders, Catholic University of Leuven, Leuven, Belgium; ^13^Baker Heart and Diabetes Institute, Melbourne, VIC, Australia; ^14^St. Olavs Hospital, Trondheim, Norway; ^15^Australian Institute for Musculoskeletal Science (AIMSS), Melbourne, VIC, Australia; ^16^School of Medical and Health Sciences, Edith Cowan University, Joondalup, WA, Australia

**Keywords:** cardiorespiratory fitness, VO_2max_, VO_2peak_, exercise training, response heterogeneity, trainability

## Abstract

There is heterogeneity in the observed 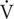
O_2peak_ response to similar exercise training, and different exercise approaches produce variable degrees of exercise response (trainability). The aim of this study was to combine data from different laboratories to compare 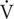
O_2peak_ trainability between various volumes of interval training and Moderate Intensity Continuous Training (MICT). For interval training, volumes were classified by the duration of total interval time. High-volume High Intensity Interval Training (HIIT) included studies that had participants complete more than 15 min of high intensity efforts per session. Low-volume HIIT/Sprint Interval Training (SIT) included studies using less than 15 min of high intensity efforts per session. In total, 677 participants across 18 aerobic exercise training interventions from eight different universities in five countries were included in the analysis. Participants had completed 3 weeks or more of either high-volume HIIT (*n* = 299), low-volume HIIT/SIT (*n* = 116), or MICT (*n* = 262) and were predominately men (*n* = 495) with a mix of healthy, elderly and clinical populations. Each training intervention improved mean 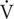
O_2peak_ at the group level (*P* < 0.001). After adjusting for covariates, high-volume HIIT had a significantly greater (*P* < 0.05) absolute 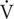
O_2peak_ increase (0.29 L/min) compared to MICT (0.20 L/min) and low-volume HIIT/SIT (0.18 L/min). Adjusted relative 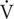
O_2peak_ increase was also significantly greater (*P* < 0.01) in high-volume HIIT (3.3 ml/kg/min) than MICT (2.4 ml/kg/min) and insignificantly greater (*P* = 0.09) than low-volume HIIT/SIT (2.5 mL/kg/min). Based on a high threshold for a likely response (technical error of measurement plus the minimal clinically important difference), high-volume HIIT had significantly more (*P* < 0.01) likely responders (31%) compared to low-volume HIIT/SIT (16%) and MICT (21%). Covariates such as age, sex, the individual study, population group, sessions per week, study duration and the average between pre and post 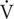
O_2peak_ explained only 17.3% of the variance in 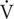
O_2peak_ trainability. In conclusion, high-volume HIIT had more likely responders to improvements in 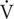
O_2peak_ compared to low-volume HIIT/SIT and MICT.

## Introduction

Health guidelines recommend aerobic exercise training for improving cardiorespiratory fitness (CRF) and reducing the risk of chronic disease and premature mortality (WHO, 2015; Ross et al., 2016). An increase of one metabolic equivalent (3.5 mL/kg/min) results in a 10–25% improvement in survival over an approximate 10-year follow-up (Blair et al., 1995; Gulati et al., 2003; Myers, 2003; Myers et al., 2011; Nes et al., 2014). There are various forms of aerobic exercise training that can be differentiated by their intensity and duration. Moderate Intensity Continuous Training (MICT) generally consists of 30–60 min of aerobic exercise at 64–76% peak heart rate (ACOS Medicine, 2017), while interval training involves more intense bouts interspersed by recovery periods (Weston et al., 2014). Interval training can be separated based on intensity into High-Intensity Interval Training (HIIT) or Sprint Interval Training (SIT). HIIT can be further defined by volume. Although classically associated with weekly loads in athletes, volume has gained acceptance to define the total duration of HIIT interval lengths (Boyd et al., 2013; Scribbans et al., 2014; Ramos et al., 2017; Eigendorf et al., 2018; Reljic et al., 2018). High-volume High-Intensity Interval Training (HIIT) typically includes repeated intervals of near maximal aerobic efforts for a specific period (e.g., 4 × 4-minute intervals at 90% peak heart rate), with a rest/recovery period in between (e.g., 3 min at 65% peak heart rate). Low-volume HIIT has fewer or shorter intervals (e.g., 6 × 1-minute intervals at 120% peak work rate) and SIT is defined as supramaximal exertion (e.g., 8 × 20-second intervals at 170% peak work rate) with active recovery/rest between intervals. Interval training has recently become popular because it is more time efficient (Phillips et al., 2017), and sometimes more enjoyable than MICT (Bartlett et al., 2011; Jung et al., 2014).

Meta-analyses have shown that high-volume HIIT is comparable, if not superior, to MICT for improving CRF (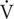
O_2max_/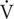
O_2peak_) and other health biomarkers (Gist et al., 2014; Weston et al., 2014; Milanovic et al., 2015; Ramos et al., 2015; Batacan et al., 2017). High-volume HIIT produces greater 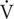
O_2max_/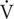
O_2peak_ changes than low-volume HIIT and SIT protocols at the group level (Bacon et al., 2013; Astorino and Schubert, 2014; Gist et al., 2014; Milanovic et al., 2015); where 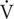
O_2max_ is a maximal effort on graded exercise test with a plateau in oxygen consumption, and 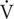
O_2peak_ is a maximal effort on a graded exercise test without a plateau in oxygen consumption (Coombes and Skinner, 2014). However, there is heterogeneity in the observed CRF response to an exercise intervention (i.e., the “trainability” of an individual). Some individuals show large improvements in CRF (often described as “responders”), whereas others show little to no-improvements (“low-responders”) following the same apparent exercise training stimulus (Bacon et al., 2013; Astorino and Schubert, 2014; Coombes and Skinner, 2014; Mann et al., 2014; Bouchard et al., 2015). Optimizing exercise training to improve CRF is imperative to long-term health; therefore, it is important to understand how factors such as the type of aerobic exercise intervention can influence observed rates of CRF trainability.

In the largest study to date on CRF trainability (The HERITAGE study; *n* = 742), 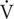
O_2max_ gain following 20 weeks of endurance training was 400 mL on average, with 7% of participants gaining 100 mL/min or less and 8% gaining 700 mL/min or more (Bouchard et al., 1999). However, this observed heterogeneity in 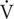
O_2max_ trainability may have resulted from technical error of measurement (TEM); a combination of random within-individual variation and/or measurement error (Atkinson and Batterham, 2015; Hecksteden et al., 2015, 2018; Hopkins, 2015; Williamson et al., 2017). Furthermore, the variability in training response should consider the minimal clinically important difference (MCID). Without considering the TEM and the MCID, identifying the probability of an individual’s response is inconclusive and CRF trainability may be misclassified (Atkinson and Batterham, 2015). Despite this, many studies to-date have used zero-change or a proportion of a group to classify “adverse-responders”, “non-responders”, “low-responders” and “high responders” to CRF training (Scharhag-Rosenberger et al., 2012; Mann et al., 2014; Gurd et al., 2016). Using this terminology based on arbitrary indicators for response is problematic and has created much debate (Montero and Lundby, 2017; Hecksteden et al., 2018). Some investigators have proposed that the concept of “non-responders” is a myth and that simply increasing the training load converts the majority of “non-responders” to “responders” (Bacon et al., 2013; Joyner, 2017; Montero and Lundby, 2017). Training load considers both the intensity and duration of exercise (Banister et al., 1992). Recently, Howden et al. (2015, 2018) found that the number of non-responders is minimal if the intensity and duration of training is high enough. Individuals were able to generate a higher stroke volume and cardiac output (Howden et al., 2015, 2018), both of which are imperative for increasing the ability of the heart to improve 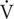
O2max (Levine, 2008).

The primary aim of this study was to utilize a large multi-center approach (*n* = 677 participants across 18 studies) to compare the number of likely responders between different training loads: high-volume HIIT, low-volume HIIT/SIT, and MICT interventions. In this study, we have taken into account the TEM and the MCID to categorize participants as either a “likely responder”, “likely non-responder”, “likely adverse responder” or “uncertain”. The use of these categories provides information on the spread of participant responses relative to the MCID. Based on the literature to date, we hypothesized that high-volume HIIT will have more likely responders compared to MICT and low-volume HIIT/SIT.

## Materials and Methods

### Participant Characteristics and Recruiting

This study includes the initial results of a larger study (PREDICT-HIIT) examining genetic predictors for 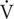
O_2peak_ trainability from HIIT/SIT and MICT interventions. Studies and potential participants were identified by contacts made through university affiliations (i.e., researchers involved in relevant studies). Studies were included if they met the following criteria: (1) participated in a HIIT, SIT, or MICT training study three or more weeks in duration within the last 15 years, (2) had an objective measure of 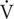
O_2peak_ (indirect calorimetry from a graded exercise test to volitional fatigue on a cycle ergometer or treadmill) before and after training, and (3) participant DNA collection was possible. Eligibility was open to male and female adults over the age of 18. Participants were included if they had greater than 80% attendance to the supervised protocol. Ethical approval was obtained from the various institutions and by the Bellberry ethical committee at the host institution (#2016-02-062-A-1).

High Intensity Interval Training (HIIT) and SIT were classified according to the intensity thresholds provided by (Weston et al., 2014). High-volume HIIT was further defined as ≥ 15 min of high-intensity efforts in total during the session and low-volume HIIT was defined as <15 min of high-intensity efforts in total during the session. SIT was classified as repeats of <1 min above maximal efforts (per bout). MICT was defined as 30 min or more of continuous exercise at 64–76% maximum heart rate (HR _max_) or equivalent. For analysis purposes, low-volume HIIT and SIT studies were combined because their training loads were similar. Training loads were based on Edwards’ training impulse (TRIMP); time in each training heart rate zone multiplied by the relative weighting factor of exercise intensity (Edwards, 1993).

### Data Analysis

Normality and homoscedasticity for 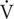
O_2peak_ response were assessed using the Shapiro–Wilk and Levene’s tests. Data are presented as mean ± standard deviation where appropriate. We used a paired sample *t*-test to calculate the group mean 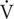
O_2peak_ response for high-volume HIIT, low-volume HIIT/SIT and MICT. Effect sizes were based on Cohen’s *d*. We used an analysis of covariance (ANCOVA) to compare adjusted relative 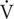
O_2peak_ responses between high-volume HIIT, low-volume HIIT/SIT and MICT. Values were adjusted for age, sex, individual study, duration of individual study, number of sessions each week, population group (e.g., coronary artery disease) and the average between pre and post-test scores (to avoid regression to the mean) (Barnett et al., 2015). The ANCOVA for absolute 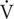
O_2peak_ also included baseline weight as a covariate. Ethnicity was not a common identifier across studies and therefore was not included as a covariate. *Post hoc* testing used Tukey’s least significance difference test. A regression analysis determined the contribution of the covariates to the 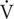
O_2peak_ response. We used an analysis of variance (ANOVA) to compare men and women group mean 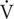
O_2peak_ responses within each intervention, and to compare 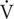
O_2peak_ responses between population groups. Statistical analyses were completed using SPSS (version 23.0, SPSS Inc., Chicago, IL, United States).

The thresholds for response categories used a combination of the TEM and the MCID. Categories included “likely responder”, “likely non-responder”, “likely adverse responder” and “uncertain”. Table 1 shows the categories illustrated with an example. Combining the MCID and TEM for the threshold improves the confidence in the “likely responder”/“non responder” classifications (e.g., compared to 2 TEM threshold (Hecksteden et al., 2018)). The TEMs for each individual study were first estimated by multiplying the mean baseline 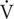
O_2peak_ value by a previously published coefficient of variation (CV) for 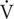
O_2peak_ of 5.6% (Katch et al., 1982). These were then averaged to obtain the TEM for each group that was used in the calculation to categorize individuals. The use of a CV of 5.6% has been suggested by others (Hecksteden et al., 2018) and is more conservative than what has been previously used (3.5%) (Edgett et al., 2018). It has been demonstrated that as little as a 1 mL/kg/min can be clinically important in individuals with coronary artery disease (Keteyian et al., 2008). Despite this, we used 3.5 mL/kg/min as the MCID based on evidence that it is associated with a 10–25% decreased risk of all-cause mortality in studies with an approximate 10-year follow-up (Blair et al., 1995; Gulati et al., 2003; Myers et al., 2011; Nes et al., 2014). A likely responder was considered a 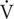
O2peak response of above one MCID plus the TEM. Individual TEMs were calculated for each study resulting in different thresholds. These individual TEMs were averaged to provide a threshold for each training intervention (high-volume HIIT, low-volume HIIT/SIT and MICT). A likely responder for the high-volume HIIT group was above 5.3 mL/kg/min, low-volume HIIT/SIT group was 5.2 mL/kg/min and MICT group was 5.0 mL/kg/min. A comparison of likely responders between interventions was calculated using Medcalc statistical software, based on the “n-1” Chi-squared test (MedCalc, 2018).

**Table 1:** Criteria for the responder categories with examples.

Category	Criteria	Example if an intervention in a study had a TEM of 5 mL/kg/min and an MCID of 3.5 mL/kg/min
Likely responder	> 1 TEM above the + MCID	> 8.5 mL/kg/min
Likely non-responder	> 1 TEM below + MCID to < 1 TEM below the −MCID	−1.5 mL/kg/min to −8.5 mL/kg/min
Likely adverse responder	> 1 TEM below the -MCID	< 8.5 mL/kg/min
Uncertain	< 1 TEM above to < 1 TEM below + MCID	−1.5 mL/kg/min to 8.5 mL/kg/min

## Results

In total, 677 participants across 18 studies from eight different universities provided data for this analysis (Table 2). These came from the University of Queensland, Australia (*n* = 191), Antwerp University and the Catholic University of Leuven, Belgium (*n* = 180), the Norwegian University of Science and Technology, Norway (*n* = 126), The Gene SMART cohort (PMID: 29143594) at Victoria University, Australia (*n* = 59), Queens University, Canada (*n* = 55), the University of British Columbia, Canada (*n* = 38), and the University of Texas Southwestern Medical Center, United States (*n* = 28).

**Table 2:** Included studies for each intervention.

	High-volume HIIT	Low-volume HIIT/SIT	MICT
University of Queensland (UQ)	Study (Ramos et al., 2016): *n* = 25 people with metabolic syndrome, 16-wk study, 4 × 4 protocol (38 min total with 16 min high intensity −10 min warm up at 60–70% HR _peak_, followed by 4x4 min at 85–90% HR_peak_, 3-min recovery in between each set at 50–70% HR _peak_), 3x/wk. **Training load per session: ¬97 AU** Study (Taylor et al., 2017): *n* = 37 people with CAD, 12-wk study, 4 × 4 protocol (38 min total with 16 min high intensity −10 min warm up at 11–13, followed by 4 × 4 min at 15–18 RPE,_,_ 3-min recovery in between each set at 11–13), 3x/wk. **Training load per session: ¬97 AU**	Study (Ramos et al., 2016): *n* = 26 people with metabolic syndrome,16-wk study, 1 × 4 protocol (17 min total with 4 min high intensity −10 min warm up at 50–60% HR _peak_, 1 × 4 min at 85–95% HR _peak_, 3 min recovery in between each set at 50–70% HR _peak_), 3x/wk. **Training load per session: ¬23.5 AU** Study (unpublished); *n* = 19 with Type-2 diabetes, 8-wk study, 1x4 protocol (26 min total with 4 min high intensity −3 min warm up and cool down at 50–60% HR _peak_,1x4 min at 85–95% HR _peak_) plus 8 × 1 min resistance exercises with 1min recovery between, RPE 17 + , 3x/wk. **Training load per session: ¬50 AU**	Study (Ramos et al., 2016): *n* = 25 people with metabolic syndrome, 16-wk study, 30 min at 60–70% HR _peak;_ 5x/wk. **Training load per session: ¬60 AU** Study (Taylor et al., 2017): *n* = 39 people with CAD, 12-wk study, 40 min at 11–13 RPE, 3x/week. **Training load per session ¬80** Study (unpublished): *n* = 20 people with Type-2 diabetes, 8-wk study, 22 min 30 s at 55–69% HR _peak_, plus 30 min of moderate intensity resistance exercises × 2/wk (8 exercises, RPE 11–13, 2 sets of 10 repetitions). **Training load per session ¬84.5** AU AND 2x/wk of MICT alone (52.5 min). **Training load per session ¬45 AU**
Antwerp University/ Catholic University of Leuven	Study (Pattyn et al., 2016): *n* = 89 people with CAD, 12-wk data; 4 × 4 protocol (38 min total with 16 min high intensity −10 min warm up at 50–70% HR _peak_, followed by 4 × 4 min at 85–95% HR_peak_, 3-min recovery in between each set at 50–70% HR _peak_), 3x/week, 3x/wk. **Training load per session: ¬97**	**N/A**	Study (Pattyn et al., 2016): *n* = 91 people with CAD, 12-week data, 47 min at 60–70% HR _peak_, 3x/week. **Training load per session: ¬94 AU**
Norwegian University of Science and Technology (NTNU)	Study (Stensvold et al., 2015): *n* = 49 seniors (70 +), 12-mth data, 4 × 4 protocol (38 min total with 16 min high intensity −10 min warm up at 60–70% HR _peak_, followed by 4 × 4 min at 85–90% HR_peak_, 3-min recovery in between each set at 50–70% HR _peak_), 3x/week. **Training load per session: ¬97 AU**	**N/A**	Study (Stensvold et al., 2015): *n* = 77 seniors, 12-month data, 50 min at 60–70% HR _peak_, 2x/wk. **Training load per session: ¬100**
Victoria University (VU) the Gene SMART cohort.	Study (Yan et al., 2017): *n* = 59 active and healthy males, 4 wk-study, up to 45 min total with 16–28 min high intensity (5 min warm up at 60 W; 8–14 × 2-min intervals at LT power + 40–70% of change in WR peak and power at LT, 1-min recovery periods at 60 W), 3x/wk. **Training load per session: ¬90- 157 AU**	**N/A**	**N/A**
Queen’s University	Study (unpublished): *n* = 12 active and healthy, 4-wk study, 4x4 protocol (38 min total with 16 min high intensity −10 min warm up at 70–75% HR _peak_, followed by 4 × 4 min at 90–95% HR_peak_, 3-min recovery in between each set at 70–75% HR _peak_), 3x/week. **Training load per session: ¬97 AU**	Studies (Boyd et al., 2013; Ma et al., 2013; Scribbans et al., 2014; Bonafiglia et al., 2016, 2017): *n* = 31 healthy participants, 4–6-wk Tabata protocol, 4 min total and up to 2 min 40 s of high intensity = 8 × 20 s sprints at 170% WR_peak_ with 10-s load-less cycling between, 4x/wk. **Training load per session: ¬20 AU** Study (Raleigh et al., 2016): *n* = 2 healthy participants, 3-wk study, up to 24 min total (8–12 × 1-min intervals at 100% WR _peak_), 4x/wk, 1 min warm-up and recovery load-less cycling between repeats. **Training load: ¬59 AU**	Study (Preobrazenski et al., 2018): *n* = 10 active and healthy adults, 4-wk study, 30 min at 65% WR _peak_, 4x/wk. **Training load per session: ¬60 AU**
University of British Columbia (UBC)	**N/A**	Study (Francois et al., 2017): *n* = 34 with Type-2 diabetes, 12-wk study, up to 25 min total exercise with up to 10 min high intensity in total (4–10 × 1 min bursts at 90% HR _max;_ 1 min recovery between, 3 min warm up and cool down), 3x/week. **Training load per session: ¬40** AU	**N/A**
		Study (Forbes et al., 2017): *n* = 4 healthy females, 3-wk study, 3 sessions each wk, up to 25 min in total, with 10 min high intensity. Session 1 = 30-sec all-out cycling sprints with 4 min recovery (progressing from 4 to 6 repeats). **Training load per session**: ¬ **32.5 AU**. Session 2 = 6-s all-out sprints with 24 sec rest (progress from 10 to 20 repeats). **Training load per session: ¬10–20 AU. S**ession 3 = 1 min sprints and recovery (progressing from 8 to 10 repeats). High intensity intervals = 85% W_max_. Recovery intervals = 15% W_max_. **Training load per session: ¬45 AU**	
University of Texas Southwestern Medical Center (UTSW)	Study (Howden et al., 2018): *n* = 28 sedentary middle-aged men and women, 2-y longitudinal study, 4 × 4 protocol^∗^ up to 2x/wk in first 6 mth and 1/wk maintenance. **Training load per session: ¬97 AU**. Base training, 30–60 min, 2 x/wk. **Training load per session: ¬60–120 AU.** MSS lactate training, 30 min, 1x/wk. **Training load per session: ¬120–150 AU**. Recovery training, 30 min, 1x/wk. **Training load per session: ¬60 AU.** Strength training 1–2 days/wk.	**N/A**	**N/A**
Total (n)	**299**	**116**	**262**

*Numbers (n), percentage (%), heart rate (HR), work rate (WR), rating of perceived exertion (RPE), maximal steady state (MSS), Watts (W), lactate threshold (LT)coronary artery disease (CAD), second (s), minutes (min), week (wk), months (mth), year, (y), repetitions (reps), 4 × 4 protocol^∗^ (38 min total with 16 min high intensity – 10 min warm up at 70–75% HR _peak_, followed by 4 × 4 min above 95% HR_peak_, 3-min recovery in between each set at 60–75% HR _peak_). Tabata protocol = 8 × 20 s sprints at 170% WR_peak_ with 10-s load-less cycling between, 4x/wk. Training load = (time in each training zone multiplied by relative weighting of exercise intensity), arbitrary units (AU).*

Participants were from various populations including those with coronary artery disease (*n* = 256), type-2 diabetes (*n* = 73), the metabolic syndrome (*n* = 76), as well as individuals who were active and healthy (*n* = 118) and individuals middle-aged or over 75 years (*n* = 154). We collated data from females (*n* = 182) and males (*n* = 495). The mean age was 56.3 ± 16.0 years.

### Relative 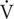
O_2peak_

**FIGURE 1:**
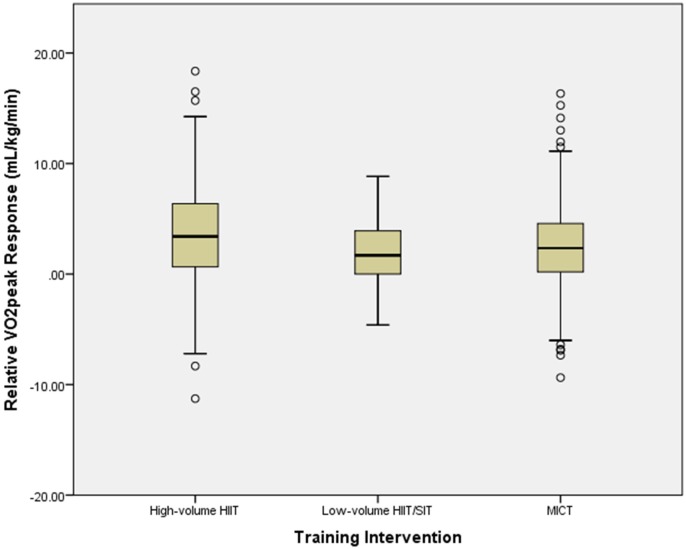
Mean relative 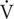
O_2peak_ response following each training intervention (raw data). Boxes contain the median (horizontal line), 25th and 75th percentile (bottom and top of box, respectively), the minimum and maximum response (bottom and top of whiskers). Individual “outliers” are dots above and below whiskers.

Table 3 and Figure 1 provide the changes in unadjusted relative 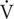
O_2peak_. Group mean relative 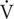
O_2peak_ scores significantly increased after all intervention types, with small effect sizes after high-volume HIIT (3.4 mL/kg/min, 95% CI 3.0 to 3.9 mL/kg/min, *P* < 0.001) Cohen’s *d* = 0.3; MICT (2.5 mL/kg/min, 95% CI 2.1 to 3.0 mL/kg/min, *P* < 0.001) Cohen’s *d* = 0.3; and low-volume HIIT/SIT (2.0 mL/kg/min, 95% CI 1.5 to 2.5 mL/kg/min, *P* < 0.001) Cohen’s *d* = 0.2. Table 4 presents the adjusted group means. A significant group difference (*P* = 0.01) was found with ANCOVA for relative 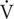
O_2peak_ response between high-volume HIIT and MICT (0.84 mL/kg/min, 95% CI 0.2 to 1.5 mL/kg/min). There was no significant difference (*P* = 0.09) between high-volume HIIT and low-volume HIIT/SIT (0.78 mL/kg/min, 95% CI −0.13 to 1.67 mL/kg/min). There was no significant difference (*P* = 0.9) between MICT and low-volume HIIT/SIT (−0.06 mL/kg/min, 95% CI −0.89 to 1.02).

**Table 3:** Baseline and relative 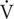
O_2peak_ response for each individual study, as well as averages for all studies combined.

Training intervention	Total *n* (% F)	Age (years)	Baseline BMI (kg/m^2^)	Pre-training 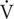 O_2peak_ (mL/kg/min)	Change (Δ) mLkg/min	Δ %	*P*-value (Cohen’s *d*)	TEM (mL/kg/min)	Likely responder *n* (%)	Uncertain *n* (%)	Likely non-responder *n* (%)	Likely adverse responder *n* (%)
**High-volume HIIT total**	**299 (22)**	**54.3 ± 16.5**	**27.0 ± 4.4**	**31.4 ± 11.0**	**3.4 ± 4.2**	**10.8**	**< 0.001 (*d* = 0.3)**	**1.8**	**92 (31)**	**98 (33)**	**105 (35)**	**4 (1)**
UQ												
(1) 4 × 4 × 3/wk, 16 wk (Ramos et al., 2016)	25 (52)	57.4 ± 1.8	32.4 ± 7.2	24.8 ± 5.0	2.9 ± 5.8	11.7	0.02 (*d* = 0.5)	1.4	9 (36)	4 (16)	11 (44)	1 (4)
(2) 4 × 4 × 3/wk, 12 wk (Taylor et al., 2017)	37 (14)	66.0 ± 6.7	28.7 ± 3.7	27.3 ± 5.7	2.6 ± 4.0	9.5	< 0.001 (*d* = 0.4)	1.5	12 (32)	8 (22)	17 (46)	0
Antwerp/Leuven 4 × 4 × 3/wk, 12-wk (Pattyn et al., 2016):	89 (8)	58.3 ± 10.0	28.0 ± 4.4	23.3 ± 5.9	4.9 ± 4.0	21.0	< 0.001 (*d* = 0.7)	1.6	38 (43)	28 (32)	21 (24)	1 (1)
NTNU												
(1) 4 × 4 × 3/wk, 1 year (Stensvold et al., 2015)	49 (39)	71.7 ± 1.8	25.3 ± 3.2	31.8 ± 6.7	3.9 ± 4.3	12.3	< 0.001 (*d* = 0.6)	1.8	17 (35)	19 (39)	11 (22)	2 (4)
VU												
(1) 8–14 × 2min × 3/wk, 4 wk (Yan et al., 2017)	59 (0)	31.0 ± 8.2	25.2 ± 3.2	46.7 ± 7.1	0.1 ± 2.7	0.2	0.053	2.6	0	20 (34)	39 (68)	0
Queen’s												
(1) 4 × 4 × 3/wk, 3wk	12 (50)	22.0 ± 2.2	25.4 ± 4.9	46.7 ± 8.6	2.5 ± 2.6	5.3	< 0.01 (*d* = 0.3)	2.6	1 (8)	7 (59)	4 (33)	0
UTS												
(1) 4 × 4 × 1/wk, base and recovery × 1–2/wk, MSS × 1/wk, strength × 2/wk, 2 years (Howden et al., 2018)	28 (54)	53.5 ± 4.8	25.6 ± 3.0	28.8 ± 5.0	5.6 ± 2.9	19.4	< 0.001 (*d* = 1.0)	1.6	15 (53)	10 (36)	3 (11)	0
**Low-volume HIIT/SIT**	**116 (43)**	**48.1 ± 18.1**	**30.3 ± 6.6**	**30.6 ± 12.8**	**2.0 ± 2.9**	**6.5**	**< 0.001 (*d* = 0.2)**	**1.7**	**18 (16)**	**37 (32)**	**61 (52)**	**0**
UQ												
(1) 1 × 4, × 3/wk, 16 wk (Ramos et al., 2016)	26 (35)	57.1 ± 7.4	31.0 ± 5.2	26.5 ± 6.3	2.3 ± 2.7	8.7	< 0.001 (*d* = 1.0)	1.5	3 (12)	10 (38)	13 (50)	0
(2) 1 × 4 + strength × 3/wk, 8 wk	19 (9)	59.5 ± 8.7	34.5 ± 6.1	21.8 ± 4.8	0.7 ± 3.1	1.8	0.4	1.2	3 (16)	1 (5)	15 (79)	0
Queens												
(1–5) 8 × 20-second sprints, 4x/wk (Boyd et al., 2013; Ma et al., 2013; Scribbans et al., 2014; Bonafiglia et al., 2016, 2017)	15 (40)	20.9 ± 1.0	24.8 ± 2.8	44.4 ± 7.1	0.6 ± 3.7	1.5	0.6	2.5	2 (13)	3 (20)	10 (67)	0
3wk	12 (0)	21.5 ± 3.7	24.4 ± 4.5	50.9 ± 9.0	3.8 ± 3.3	7.5	< 0.01 (*d* = 0.4)	2.9	3 (25)	7 (58)	2 (17)	0
4wk	4 (0)	22.0 ± 1.2	25.2 ± 1.5	47.3 ± 6.2	3.1 ± 2.7	6.6	0.1	2.8	1 (25)	3 (75)	0	0
6wk	2 (50)	21.5 ± 2.1	34.6 ± 2.5	35.0 ± 1.8	1.6 ± 1.7	4.6	0.4	1.9	0 (0)	1 (50)	1 (50)	0
(6) 8–12 × 1 min intervals × 4/wk, 3 wk (Raleigh et al., 2016)												
UBC												
(1) 4–10 × 1 min × 3/wk, 12 wk (Francois et al., 2017)	34 (69)	55.3 ± 13.6	33.4 ± 6.6	22.1 ± 7.3	2.2 ± 2.0	6.6	< 0.001 (*d* = 0.3)	1.2	4 (12)	8 (24)	22 (64)	0
(2) Up to 1 min intervals × 3/wk, 3 wk (Forbes et al., 2017)	4 (100)	21.5 ± 4.4	NA	40.1 ± 7.7	3.6 ± 3.0	8.9	0.1	2.2	1 (25)	2 (50)	1 (25)	0
**MICT**	**262 (26)**	**62.0 ± 12.1**	**27.6 ± 5.3**	**27.5 ± 8.1**	**2.5 ± 3.8**	**9.1**	**< 0.001 (*d* = 0.3)**	**1.5**	**55 (21)**	**89 (34)**	**110 (42)**	**8 (3)**
UQ												
(1) 30 min × 5/wk, 16 wk (Ramos et al., 2016)	25 (32)	54.5 ± 9.6	32.5 ± 6.0	27.5 ± 8.0	1.4 ± 5.6	5.1	0.2	1.5	4 (16)	5 (20)	13 (52)	3 (12)
(2) 40 min × 3/wk, 12 wk (Taylor et al., 2017)	39 (18)	65.3 ± 6.8	26.9 ± 2.3	27.4 ± 7.5	1.9 ± 4.0	6.9	< 0.01 (*d* = 0.2)	1.5	7 (18)	11 (28)	19 (49)	2 (5)
(3) 22.5 min + 30 min strength × 2/wk and 52.5 min × 2/wk, 8 wk	20 (8)	60.5 ± 7.0	30.6 ± 10.2	25.4 ± 6.6	0.2 ± 2.0	0.8	0.7	1.4	0	3 (15)	17 (85)	0
Antwerp/Leuven												
(1) 47 min × 3/wk, 12 wk (Pattyn et al., 2016)	91 (10)	57.9 ± 8.7	28.3 ± 4.3	22.7 ± 5.6	4.3 ± 3.25	18.9	< 0.001 (*d* = 0.7)	1.3	33 (36)	34 (38)	24 (27)	0
NTNU												
(1) 50 min × 2/wk, 12 mth (Stensvold et al., 2015)	77 (45)	72.5 ± 2.1	24.7 ± 2.9	31.1 ± 5.9	1.5 ± 3.4	4.8	< 0.001 (*d* = 0.2)	1.7	10 (13)	28 (36)	36 (47)	3 (4)
Queens												
(1) 30 min × 4/wk, 4wk	10 (0)	23.1 ± 5.3	25.9 ± 4.4	47.2 ± 5.7	4.0 ± 2.2	8.5	< 0.001 (*d* = 0.8)	2.6	2 (20)	7 (70)	1 (10)	0
**Total**	**677 (27)**	**56.3 ± 16.0**	**27.8 ± 5.3**	**29.7 ± 10.5**	**2.8 ± 3.9**	**9.4**	**< 0.001 (*d* = 0.3)**	**1.7**	**162 (24)**	**229 (34)**	**274 (40)**	**12 (2)**

*^∗^Values shown are mean ± standard deviation (SD). Number of participants (n), percentage (%), body mass index (BMI), 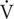
O_2peak_ (peak aerobic fitness), weeks (wk), minutes (min), technical error of measurement (TEM), female (F), maximal steady state (MSS). ^∗∗^The TEM for each individual study was different to the average TEM for each of the three training groups (high-volume HIIT, low-volume HIIT/SIT and MICT).*

### Absolute 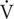
O_2peak_

Absolute 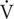
O_2peak_ values were significantly increased after all intervention types (Table 4). There were small effect sizes after high-volume HIIT (0.27 L/min, 95% CI −0.33 to –0.23, *P* < 0.001) Cohen’s *d* = 0.3; MICT (0.19 L/min, 95% CI −0.24 to −0.12, *P* < 0.001) Cohen’s *d* = 0.2; and low-volume HIIT/SIT (0.16 L/min, 95% CI −0.22 to −0.10 to, *P* < 0.001) Cohen’s *d* = 0.2. Table 4 presents the adjusted group means. Small significant group differences were found with ANCOVA for the change in absolute 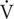
O_2peak_ between high-volume and low-volume HIIT/SIT (0.12 L/min, 95% CI 0.02 to 0.22 L/min, *P* < 0.05) and between high-volume HIIT and MICT (0.09 mL/kg/min, 95% CI 0.02 to 0.17 L/min, *P* = 0.01). There was no significant difference (*P* = 0.61) between MICT and low-volume HIIT/SIT (−0.02 L/min, 95% CI −0.13 to 0.08 L/min).

### Different Populations

There was a significant difference between the increases in men and women’s absolute 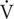
O_2peak_ values in high-volume HIIT. Men had a greater (*P* < 0.01) increase (0.49 L/min, 95% CI 0.26 to 0.38) compared to women (0.15 L/min, 95% CI 0.09 to 0.21). There were no other significant differences between men and women’s relative and absolute 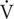
O_2peak_ responses to high-volume HIIT, low-volume HIIT/SIT or MICT.

Table 5 shows that when analyzed according to the population type, middle-aged and elderly participants had a significantly greater (*P* < 0.001) increase in relative 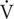
O_2peak_ with high-volume HIIT than MICT. Young and healthy participants responded significantly more favorably (*P* < 0.05) to MICT than low-volume HIIT/SIT and high-volume HIIT. Participants with coronary artery disease and middle-aged and elderly participants in the high-volume HIIT group had a 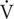
O_2peak_ response greater than the MCID. Those with coronary artery disease and young and healthy participants also had a 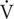
O_2peak_ response greater than the MCID with MICT. All other population groups and training interventions failed to reach the MCID.

**Table 4:** Adjusted means for absolute and relative 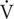
O_2peak_ response.

	Relative mean		Absolute	
Intervention	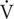 O_2peak_ increase^∗^		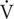 O_2peak_ increase^∗∗^	
	mL/kg/min ± SD	95% CI	L/min ± SD	95% CI
High-volume HIIT	3.3 ± 3.7	2.9–3.7	0.29 ± 0.40	0.25–0.34
Low-volume HIIT	2.5 ± 4.1	1.7–3.3	0.18 ± 0.44	0.09–0.26
MICT	2.4 ± 3.8	2.0–2.9	0.20 ± 0.42	0.15–0.26

*^∗^Adjusted for sex, age, individual study, study duration, sessions per week, population group and the average between pre and post-test scores. ^∗∗^Adjusted for sex, age, individual study, study duration, sessions per week, population group, baseline weight, and the average between pre and post-test scores and the pre weight.*

**Table 5:** 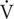
O_2peak_ response in different population groups.

	Relative 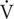 O_2peak_		
Population	increase (mL/kg/min ± SD)		
	High-volume	Low-volume	
	HIIT	HIIT/SIT	MICT
Coronary artery disease	4.19 ± 4.12	NA	3.59 ± 3.66
Type II diabetes and/or metabolic syndrome	2.73 ± 4.13	1.86 ± 4.07	0.95 ± 4.01
Middle-aged and elderly	4.50 ± 3.93ˆ	NA	1.50 ± 3.36
Young and healthy	1.10 ± 3.11^∗^	2.28 ± 3.53	4.02 ± 2.23

*Not assessed due to no participants (NA). Significantly different to MICT ^∗^P < 0.05, ˆP < 0.001.*

Overall, the covariates examined (sex, age, individual study, study duration, sessions per week, population group and the average between pre and post-test scores) contributed to 17.3% of the change in relative 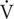
O_2peak_ (*P* < 0.001). Individual studies had the largest impact, explaining 13.5% of 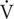
O_2peak_ response (*P* < 0.001).

### Categories of 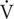
O_2peak_ Responders

**FIGURE 2:**
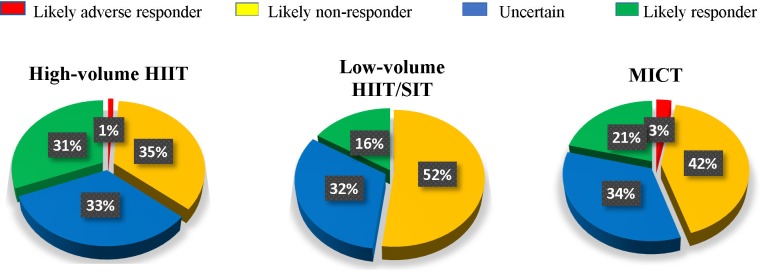
Percentage of likely responders to changes in relative 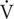
O_2peak_ in each training intervention.

Table 3 and Figure 2 outlines the thresholds and percentages of likely responders, likely non-responders, likely adverse responders and those uncertain (not classified as a likely responder or likely non-responder) for each training intervention, and the individual studies contributing to these training interventions. High-volume HIIT had significantly more likely responders (31%) compared to MICT (21%) and low-volume HIIT/SIT (16%), *P* < 0.01. There were comparable responders classified as uncertain (∼33%) across high-volume HIIT, low-volume HIIT/SIT and MICT. On average, high-volume HIIT had a greater training load (∼100 Arbitrary Units (AU)) compared to low-volume HIIT/SIT (∼33 AU) and MICT (∼75 AU).

Studies with short durations (3–4 weeks) had fewer likely responders (13% average) irrespective of whether the intervention was high-volume HIIT, low-volume HIIT/SIT or MICT (Boyd et al., 2013; Ma et al., 2013; Scribbans et al., 2014; Bonafiglia et al., 2016, 2017; Raleigh et al., 2016; Yan et al., 2017). Participants from the shorter duration studies were younger (mean - 23.1 years) and had a higher 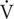
O_2peak_ (44.4 mL/kg/min) prior to the intervention (Stensvold et al., 2015; Howden et al., 2018). From the individual studies over 4 weeks in duration, the study (Howden et al., 2018) with the most likely responders (53%) had the greatest average training load per session (up to ∼ 150 AU), the longest-running intervention (2 years) and the most training sessions each week (up to five). Most studies had three training sessions per week with 28% of participants classified as a likely responder (Stensvold et al., 2015; Pattyn et al., 2016; Ramos et al., 2016; Forbes et al., 2017; Francois et al., 2017; Yan et al., 2017). One study had two sessions each week, with 13% of participants classified as a likely responder (Stensvold et al., 2015).

Studies with middle-aged (50–60 years) and elderly participants (70+ years) had significantly (*P* < 0.001) more likely responders with high-volume HIIT (44% average) compared to MICT (13% average) (Stensvold et al., 2015; Howden et al., 2018). Studies with coronary artery disease participants had comparable likely responders with high-volume HIIT (38% average) and MICT (27% average), *P* = 0.06 (Pattyn et al., 2016; Taylor et al., 2017). Those with metabolic syndrome and/or type-2 diabetes (Ramos et al., 2016; Francois et al., 2017) had significantly more likely responders with high-volume HIIT (34% average) compared to low-volume HIIT/SIT (17% average) and MICT (8% average), *P* < 0.001. Likely adverse responders were from studies with elderly participants; three participants came from a MICT intervention (Stensvold et al., 2015) and two participants came from a high-volume HIIT intervention (Stensvold et al., 2015). Likely adverse responders also included one participant with coronary artery disease from a high-volume HIIT intervention and two from a MICT intervention (Pattyn et al., 2016), and one participant with metabolic syndrome from a high-volume HIIT intervention and 1 from a MICT intervention (Ramos et al., 2016).

## Discussion

Establishing a dose (i.e., intensity, frequency and duration) of exercise training that improves the number of observed responders with a clinically meaningful improvement to exercise training may reduce the prevalence of chronic disease risk and all-cause mortality associated with a low cardiorespiratory fitness. However, much of the research-to-date has focused largely on group-mean changes, with minimal studies comparing interventions and clinically meaningful responses. Furthermore, many of these studies have been small in sample size (between 10 and 20 participants), with potentially large variations in participant baseline physical activity levels, and training responses that are statistically underpowered. With personalized medicine becoming increasingly widespread, the aim of this study was to compare, in a relatively large sample size (*n* = 677) from different laboratories, the observed rates of likely responders between a variety of aerobic training interventions. Our study adds to the current literature by showing high-volume HIIT has significantly more likely responders compared to low-volume HIIT/SIT and MICT.

**FIGURE 3:**
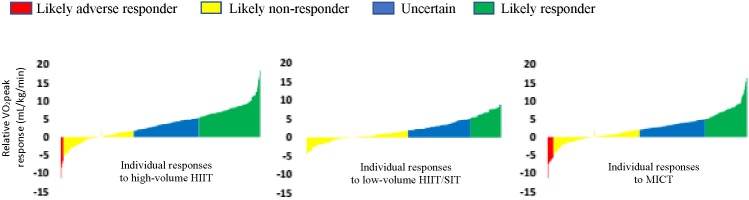
Waterfall plots of the relative 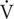
O_2peak_ (mL/kg/min) response rates for each intervention (raw data).

Meta-analyses have shown that high-volume HIIT is comparable, if not superior, to MICT for improving CRF (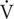
O_2max_/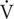
O_2peak_) and other health biomarkers (Gist et al., 2014; Weston et al., 2014; Milanovic et al., 2015; Ramos et al., 2015; Batacan et al., 2017). The group mean changes from this study were similar to previous research indicating that high-volume HIIT had a larger mean 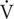
O_2peak_ gain than MICT and low-volume HIIT/SIT and studies with the greatest 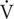
O_2peak_ gains used longer high intensity intervals/high-volume HIIT (Bacon et al., 2013). Despite these group mean changes, there was considerable heterogeneity in 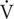
O_2peak_ responses in each intervention (Figure 3). An approach to assess whether the inter-individual training response is true that has gained much support involves comparing the adjusted standard deviations between the training group and a control comparator group (Atkinson and Batterham, 2015; Williamson et al., 2017). If the standard deviation of the training group is clinically significant and larger than the control group, it can be assumed that a true individual response has occurred. Because our data did not include a control comparator group, we felt this approach was not warranted. Nonetheless, when looking at the standard deviation changes of the adjusted group means, they were all greater than the MCID (3.5 mL/kg/min). Overall, the number of “responders” for each intervention was slightly lower in comparison to previous reports (Scharhag-Rosenberger et al., 2012; Gurd et al., 2016; Hecksteden et al., 2018). However, many studies to-date have based thresholds for response on a percentage of change or one TEM away from zero; a lower threshold will produce more “responders” (Scharhag-Rosenberger et al., 2012; Gurd et al., 2016; Hecksteden et al., 2018). Furthermore, these studies have predominantly examined healthy but sedentary populations, whereas our data had a mix of clinical and healthy populations (Timmons et al., 2010; Scharhag-Rosenberger et al., 2012; Gurd et al., 2016; Hecksteden et al., 2018). For example, our data demonstrated that participants with coronary artery disease and middle-aged and elderly participants in the high-volume HIIT group had a 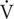
O_2peak_ response greater than the MCID. Those with coronary artery disease and young and healthy participants also had a 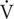
O_2peak_ response greater than the MCID with MICT. All other population groups and training interventions failed to reach the MCID. Middle-aged adults and the elderly, participants with type II diabetes and/or metabolic syndrome had a greater proportion of responders with high-volume HIIT; whereas response rates between exercise training loads were similar in participants with coronary artery disease and those who were young and healthy. There were 4 participants from our data that were classified as “likely adverse responders”; rather than a true adverse response, these participants may have performed poorly on the testing day.

It has been argued that some people are “dose-sensitive” as opposed to a “non-responder” (Montero and Lundby, 2017; Williamson et al., 2017). If physiological systems are maximized, it seems possible that everyone can improve their 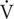
O_2peak_ (Bacon et al., 2013; Joyner, 2017; Montero and Lundby, 2017). A clinically meaningful 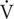
O_2peak_ response is unlikely if maximal stroke volume, oxygen transport and oxygen utilization does not improve (Sarzynski et al., 2017). Furthermore, exercise can alter the expression of genes related to mitochondrial function and energy use (Barres et al., 2012; Denham et al., 2015). Methylation can increase gene expression and affect metabolic adaptions in skeletal muscle (Barres et al., 2012). In skeletal muscle, most genes related to metabolism are demethylated following long-term exercise training (Voisin et al., 2014). These changes appear to be dose dependent and transient, with higher intensity exercise (80% heart rate maximum) causing greater demethylation and gene expression compared to lower-intensity exercise (40% heart rate maximum) where the total volume of exercise (caloric expenditure) is similar (Barres et al., 2012). To summarize, a higher training load may be more effective in those “dose sensitive” or those considered a “low responder” to training because participants are working at a threshold high enough to activate certain genes and molecular pathways required to induce a clinically meaningful exercise training response (Mann et al., 2014). Our analysis demonstrated that studies with the longest duration intervention and highest overall training loads produced the greatest 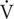
O_2peak_ gains and more likely responders (Stensvold et al., 2015; Pattyn et al., 2016; Howden et al., 2018). Typically these studies included high-volume HIIT. Our results complement previous research that indicates a greater training load correlates with fewer non-responders. For example, a recent study on 78 young males found that when training was increased from 60 to 180 min to 240–300 min per week, the number of responders (1 × technical error of measurement relative to zero) increased from 30–71 to 100%, respectively, (Montero and Lundby, 2017). It would be interesting to see if those who were deemed a “likely non-responder” from our analysis would “respond” with an increase in training duration, frequency or intensity.

Our analysis showed that age, sex, the individual study, study duration, number of sessions each week, the population group and the average between pre and post-test scores predicted 17.3% of the variance in training response; with the individual study being the highest predictor (13.5%) for 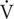
O_2peak_ response. This suggests there were other more substantial factors that affected 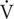
O_2peak_ trainability. In the HERITAGE study, 15% of the variation in the response to MICT was attributed to baseline 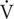
O_2peak_, age, sex, body mass and ethnicity combined; with approximately 6% attributed to workload fluctuations, 20% to technical error and daily changes, and up to 50% to genetic make-up (Sarzynski et al., 2017). A systematic review from our group identified 97 genetic variants that have been associated with 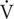
O_2peak_ trainability (Williams et al., 2017). It would be interesting to explore if those classified as a likely non-responder within our study have common genetic variants that may contribute to them being “dose sensitive”. This will be investigated in our PREDICT-HIIT Study by combining the 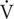
O_2peak_ data presented here with genetic analyses.

Although there were several limitations to our study, the heterogeneity of the participants and training approaches should improve external ecological validity. Combining meaningful data from small, individual studies, as we have presented here, is necessary if we seek robust, reproducible, and translational results in exercise science (Eynon et al., 2017). Data was collated from 18 different studies with different protocols and equipment for testing. Participants were predominantly males, training status varied between studies (active vs. sedentary populations), and there was a mixture of clinical (CAD, diabetes, metabolic syndrome) and healthy populations. The age (between groups, 18–81 years), volume of work (60 min to 4 min and 50% heart rate peak to 170% work rate peak) and overall duration (3 to 104 weeks) varied considerably for the individual studies included in the current analysis. These factors are very likely to contribute to training response. Furthermore, some of the individual studies did not control for variables like diet, medication use, smoking status, sleep and recovery time. Lack of sleep or poor nutrition may negatively affect the intensity an individual can train and how fast they can recover between sessions; possibly combining to reduce training response through several interactions, such as genetic and epigenetic changes (Timmons, 2011; Voisin et al., 2014; Hecksteden et al., 2015; Paul et al., 2015; Yan et al., 2016). Our TEM was calculated using one that has been previously published (Katch et al., 1982). A more robust approach is to measure an individual’s 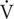
O_2peak_ response in a test-retest study (Hopkins, 2000) or with a time-matched control group. This information was not collected for each individual intervention from our study. We also acknowledge that our research focuses on several select studies and represents a small portion of MICT, HIIT and SIT related literature. Finally, adherence to the exercise training prescription has been found to impact on studies comparing HIIT to MICT (Pattyn et al., 2016; Ellingsen et al., 2017). In these studies people allocated to the HIIT group did not meet the target exercise intensities and those in the MICT group trained at a higher intensity. In our analysis we have not taken this into account and used an intention to treat analysis approach with the belief that it would be more externally valid.

Future research with cross-over designs will determine if a participant may have a better response to an alternative intervention. Such a design is costly and seldom used but potentially decreases the random variation that may occur from comparing just one pre and post-test score, and measures how an individual will respond to different training interventions (Hecksteden et al., 2015). A recent cross over study (Bonafiglia et al., 2016) compared the number of responders to SIT with MICT. Participants (*n* = 21) had to complete four sessions a week of SIT or MICT (separated by a 3-month washout period). Both interventions produced similar group mean changes in 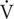
O_2peak_, and similar rates of response (based on 2 × TEM). Some individuals responded to MICT, but not to SIT and vice versa; whereas others did not improve their 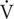
O_2peak_ in either intervention (Bonafiglia et al., 2016). Thus, those who fail to have a clinically meaningful 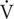
O_2peak_ response to an exercise training approach within our study may benefit from another form of training.

In conclusion, high-volume HIIT had a greater average training load and significantly more likely responders compared to low-volume HIIT/SIT and MICT. Individual studies with the smallest duration and training loads generally had the least significant gains and fewer clinically meaningful 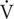
O_2peak_ responders. Future large, well-controlled studies with comparator groups and cross-over designs may help to identify influential variables and the ideal training load for 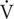
O_2peak_ trainability.

## Data Availability

The raw data supporting the conclusions of this manuscript will be made available by the authors, without undue reservation, to any qualified researcher.

## Author Contributions

CW, JC, and NE contributed to the conception and design of the study. CW organized the database and wrote the first draft of the manuscript. CW, SV, and ZL performed the statistical analysis. NH, IC, NH, JT, TG, JR, RF, JL, MF, BG, JB, CH, SS, SK, SJ, EVC, PB, VC, NP, EH, UW, AB, DS, DB, IP, and XY were investigators involved with the studies used in analysis and assisted with data collation. All authors contributed to manuscript revision, read, and approved the submitted version.

## Conflict of Interest Statement

The authors declare that the research was conducted in the absence of any commercial or financial relationships that could be construed as a potential conflict of interest.
